# Fusion Imaging of Non-Invasive and Invasive Cardiac Electroanatomic Mapping in Patients with Ventricular Ectopic Beats: A Feasibility Analysis in a Case Series

**DOI:** 10.3390/diagnostics14060622

**Published:** 2024-03-15

**Authors:** Matilda Muça, Stepan Zubarev, Dirk Bastian, Janusch Walaschek, Veronica Buia, Harald Rittger, Arsenii Dokuchaev, Thomas Bayer, Laura Vitali-Serdoz

**Affiliations:** 1 Division of Cardiac Electrophysiology, Department for Cardiology, Klinikum Fuerth, 90766 Fuerth, Germany; matilda02@hotmail.it (M.M.); zubarevstepan@gmail.com (S.Z.); dirk.bastian@klinikum-fuerth.de (D.B.); janusch.walaschek@klinikum-fuerth.de (J.W.); veronica.buia@klinikum-fuerth.de (V.B.); harald.rittger@klinikum-fuerth.de (H.R.); zodelheim@gmail.com (A.D.); 2 Radiology Department, Klinikum Fuerth, 90766 Fuerth, Germany; thomas.bayer@klinikum-fuerth.de

**Keywords:** non-invasive electrocardiographic imaging, ventricular ectopic beats, electroanatomic mapping, fusion imaging

## Abstract

In patients with premature ventricular contractions (PVCs), non-invasive mapping could locate the PVCs’ origin on a personalized 3-dimensional (3D) heart model and, thus, facilitate catheter ablation therapy planning. The aim of our report is to evaluate its accuracy compared to invasive mapping in terms of assessing the PVCs’ early activation zone (EAZ). For this purpose, non-invasive electrocardiographic imaging (ECGI) was performed using the Amycard 01C system (EP Solutions SA, Switzerland) in three cases. In the first step, a multichannel ECG (up to 224 electrodes) was recorded, and the dominant PVCs were registered. Afterward, a cardiac computed tomography (in two cases) or magnetic resonance imaging (in one case) investigation was carried out acquiring non-contrast torso scans for 8-electrode strip visualization and contrast heart acquisition. For the reconstructed epi/endocardial meshes of the heart, non-invasive isochronal maps were generated for the selected multichannel ECG fragments. Then, the patients underwent an invasive electrophysiological study, and the PVCs’ activation was evaluated by a 3D mapping system (EnSite NavX Precision, Abbott). Finally, using custom-written software, we performed 3D fusion of the non-invasive and invasive models and compared the resulting isochronal maps. A qualitative analysis in each case showed the same early localization of the dominant PVC on the endocardial surface when comparing the non-invasive and invasive isochronal maps. The distance from the EAZ to the mitral or tricuspid annulus was comparable in the invasive/non-invasive data (36/41 mm in case N1, 73/75 mm in case N2, 9/12 mm in case N3). The area of EAZ was also similar between the invasive/non-invasive maps (4.3/4.5 cm^2^ in case N1, 7.1/7.0 cm^2^ in case N2, 0.4/0.6 cm^2^ in case N3). The distances from the non-invasive to invasive earliest activation site were 4 mm in case N1, 7 mm in case N2, and 4 mm in case N3. Such results were appropriate to trust the clinical value of the preoperative data in these cases. In conclusion, the non-invasive identification of PVCs before an invasive electrophysiological study can guide clinical and interventional decisions, demonstrating appropriate accuracy in the estimation of focus origin.

## 1. Introduction

The identification of a ventricular arrhythmia’s origin may be difficult using a standard 12-lead electrocardiogram (ECG), especially if it arises in complex anatomic sites [1,2,3]. However, its accurate localization is relevant not only for the ablation therapy outcome, but also because it can influence the procedural time and the radiation exposure.

Non-invasive electrocardiographic imaging (ECGI, Brussels, Belgium) is a modern technology whose first application in humans was demonstrated in the seminal articles of Rudy Y et al. [4]. Since then, there has been rapid growth in interest and, as a consequence, emergence of different commercial systems [5,6,7,8,9,10,11]. Despite a number of differences (total number of electrodes used, compatibility with different types of imaging methodologies) most known systems have a similar operating principle. It implies the combined integration of non-invasive electrocardiographic recordings and cardiac computer tomographic (cCT) or magnetic resonance imaging (cMRI) data. One of the systems registered on the market and installed in our clinic is the Amycard 01C (EP Solutions SA, Yverdon-les-Bains, Switzerland). The accuracy of the Amycard 01C technology has been described in patients with implanted pacemakers [7,12,13] via invasive stimulation using an invasive 3-dimensional (3D) CARTO mapping system [7] and with ventricular arrhythmias [14,15,16,17,18].

In the present three-case series, we compare the non-invasive and invasive 3D models to qualitatively and quantitatively evaluate the concordance between the two technologies (Amycard 01C and EnSite NavX Precision, Abbott, Chicago, IL, USA) in identifying the early activation zone (EAZ) in patients with premature ventricular contractions (PVCs).

## 2. Case Presentation

### 2.1. Case N1

**Figure 1:**
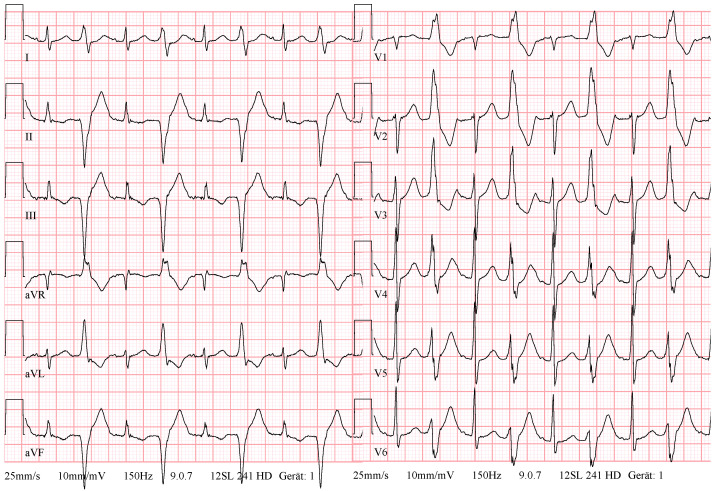
Case N1. Sinus rhythm, monomorphic premature ventricular contractions with bigeminy pattern.

A patient with ischemic cardiomyopathy and previous multiple coronary angioplasties was referred for the treatment of symptomatic monomorphic PVCs (burden 20–50%). A transthoracic echocardiography showed reduced systolic function of the left ventricle (LV). The 12-lead ECG demonstrated sinus rhythm and frequent monomorphic PVCs with a superior axis and a right bundle branch block pattern, with R/S transition in the V4-V5 on the precordial leads (Figure 1).

Other characteristics of the arrhythmia are presented in Table 1.

**Figure 2:**
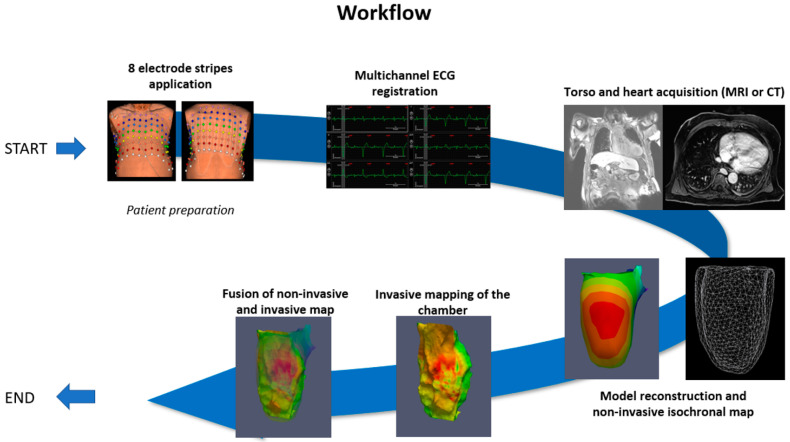
Schematic representation of the workflow, starting from the electrocardiographic (ECG) registration, proceeding to imaging and 3D segmentation, to invasive mapping, and, finally, to the fusion imaging of non-invasive and invasive data.

The workflow of the current study included several steps (Figure 2).

In the first step, multichannel ECG registration during sinus rhythm on the Amycard 01C system (EP Solutions SA, Yverdon-les-Bains, Switzerland) was performed. Dedicated 8-electrode strips compatible with cCT were placed on the patient’s torso. In total, 26 strips corresponding to 208 unipolar body surface metal ECG electrodes were used. The number of strips and unipolar body-surface electrodes implemented for each patient is shown in Table 2.

Typical clinical PVCs were registered and captured. The anonymized ECG tracings with PVCs were saved in ‘ecg.240’ format for further analysis.

**Figure 3:**
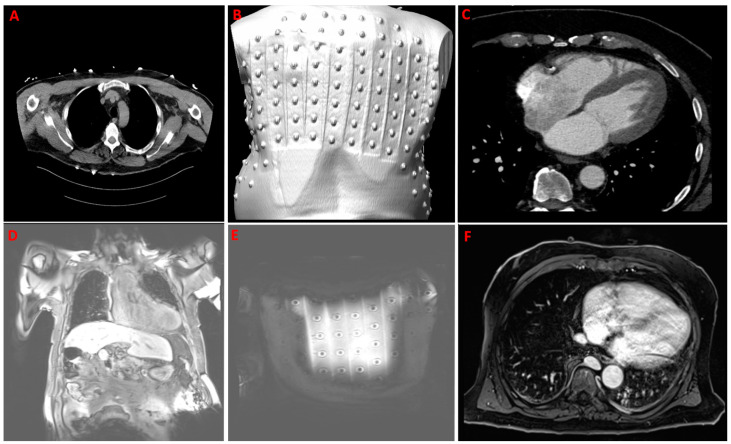
Upper row: example of recording computer tomography (CT). (**A**,**B**)—CT torso acquisition to capture applied metal 8-electrode strips (‘LungLowDose’ scan without contrast); (**C**)—contrast CT heart acquisition. Lower row: example of recording magnetic resonance imaging (MRI). (**D**,**E**)—torso series (t1_vibe_fs_cor_bh) in coronal plane to capture applied carbon MRI-compatible 8-electrode strips; (**F**)—MRI contrast heart acquisition with t1_vibe_fs_tra_p2_bh_iso_320 sequence.

In the second step, the patient underwent cCT (Somatom Definition Edge 126, Siemens AG, Munich, Germany). A low-dose thorax scan (‘LungLowDose’ without contrast and a thickness equal to 2 mm), as a first series, helped to identify the anatomical localization of the metal body-surface electrodes and the individual torso anatomy (Figure 3A,B). An ECG-gated contrast scan of the heart with a thickness equal to 0.75 mm (Figure 3C), as a second series, was achieved using automated intravenous injection of a non-ionic contrast (Solutrast 370—100 mL) during a breath hold. A retrospective acquisition protocol was used. The tomography data were exported in Dicom format.

**Figure 4:**
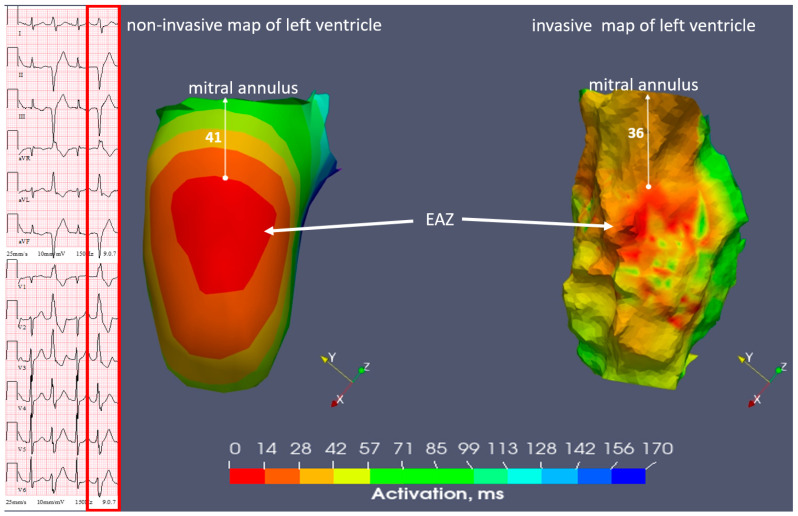
Case N1. Sinus rhythm, premature ventricular contraction. Three-dimensional representation of the non-invasive isochronal map on the left, and of the invasive isochronal map on the right. EAZ—early activation zone (red color). Approximately 41 mm distance from EAZ to mitral annulus on non-invasive isochronal map; 36 mm distance from EAZ to mitral annulus on invasive isochronal map.

In the third step, an electroanatomic mesh was reconstructed, and non-invasive isochronal maps of the ventricles were generated using the multichannel ECG and the cCT data. Model reconstruction (with 597 LV mesh nodes) and the creation of isochronal maps were carried out by dedicated software version 2.3.0 (Amycard 01C, EP Solutions SA, Yverdon-les-Bains, Switzerland). Subsequently, the EAZ was evaluated. This zone corresponded to the red-colored area on the automatically generated endocardial non-invasive isochronal map, and a white marker was added to point out the earliest activation site inside the EAZ. Afterwards, a qualitative assessment of the location of the EAZ during a typical PVC was performed. The quantitative analysis included two types of measurements. Calculation of the distance from the border of the EAZ (red color) to the atrioventricular annulus, as demonstrated in Figure 4, was the first measure. A measurement of the EAZ area (red color) was the second one.

In the fourth step, the invasive analysis was obtained by a point-by-point endocardial activation mapping of the LV, with a total of 256 points, using the EnSite NavX Precision system (Abbott) with an irrigated deflectable contact-force ablation catheter (TactiCath SE, Abbott) and with a high-density multipolar mapping catheter (Advisor^TM^ HD-Grid, Abbott). Mapping of LV was performed after transseptal puncture. Invasively, the EAZ position was identified qualitatively and quantitatively as the earliest endocardial activation during the PVCs (i.e., local bipolar ventricular electrograms before the onset of the surface QRS complex and a QS complex on unipolar recordings). In addition, a quantitative analysis using the same metrics as the non-invasive mapping was performed.

The non-invasive and invasive isochronal maps revealed the earliest activation of the dominant PVC on the endocardial surface of the inferior wall of the LV. This area extended to the midventricular inferior wall, near the base of the posteromedial papillary muscle. After identifying the PVCs’ origin point, radiofrequency energy was delivered using a power of 30 to 40 W with a temperature not exceeding 42 °C degrees. Radiofrequency ablation was performed in the early activation area, abolishing the clinical PVCs. A recorded invasive endocardial model with an isochronal map was extracted in text format from the EnSite NavX Precision system.

**Figure 5:**
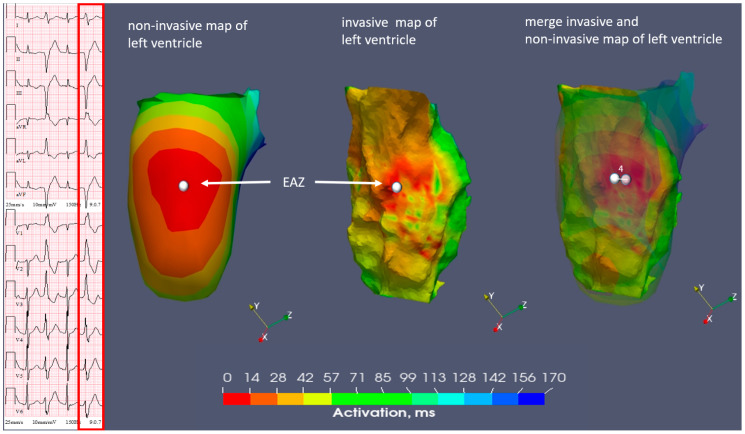
Case N1. Sinus rhythm, premature ventricular contraction. Three-dimensional representation of the endocardial non-invasive isochronal map on the left, of the invasive isochronal map in the middle, and the fusion of non-invasive and invasive maps on the right. EAZ—early activation zone. White marker—the earliest activation site of EAZ. Approximately 4 mm distance between non-invasive and invasive earliest activation site on fusion model.

In the fifth step, extraction and further import of the non-invasive and invasive models in text format into a custom written software in Python version 3.9 was performed in the same manner as in the previous study of Zubarev et al. [19]. In this step, 3D fusion was created based on combining the non-invasive and invasive models with isochronal maps using an iterative nearest point algorithm. Finally, in the merged model, the distance between the earliest activation site labeled with the white marker on both the non-invasive and invasive map, respectively, was measured, as in Figure 5.

The distance from the EAZ to the annulus was 36 millimeters (mm) versus 41 mm on the invasive and non-invasive mapping, respectively (Figure 4). The area of the EAZ was also similar between the invasive/non-invasive maps (4.3 versus 4.5 cm^2^) (Table 3).

The distance between the earliest activation site on the invasive and non-invasive isochronal maps equaled 4 mm (Figure 5).

The qualitative and quantitative analysis results are summarized in Table 3.

### 2.2. Case N2

A patient without known structural cardiomyopathy was referred to our center, presenting a long history of highly symptomatic PVCs with a burden up to 30% during Holter monitoring. A transthoracic echocardiography showed preserved systolic function of the LV. Antiarrhythmic therapy with a beta-blocker and additional flecainide and subsequent administration of propafenone were able to reduce but not able to suppress the PVCs (burden after therapy 15–20%).

**Figure 6:**
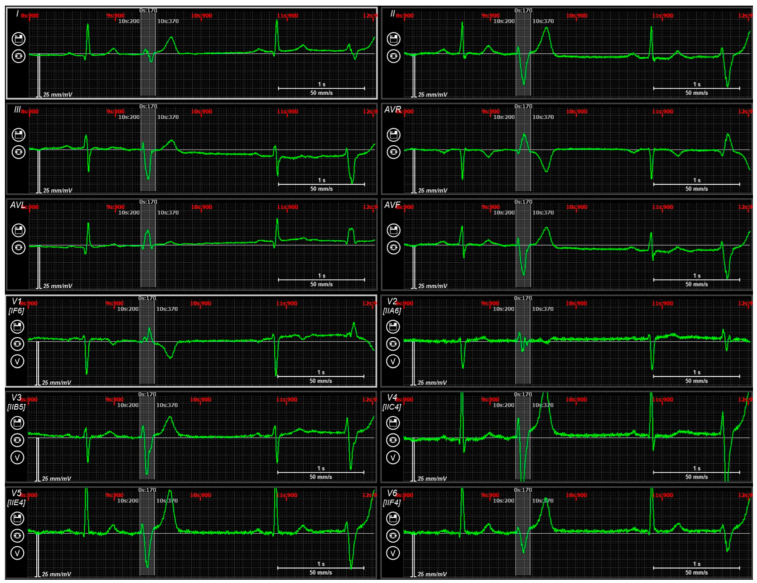
Case N2. Sinus rhythm, monomorphic premature ventricular contractions with bigeminy pattern and right bundle branch morphology.

The 12-lead ECG demonstrated sinus rhythm and frequent monomorphic PVCs with a superior axis, right bundle branch type of morphology, with R/S transition in the V2-V3 precordial leads (Figure 6).

The characteristics of the arrhythmia are presented in Table 1.

In case N2, 28 strips corresponding to 224 unipolar body surface MRI-compatible carbon electrodes were placed onto the patient’s torso and used for ECG recording during sinus rhythm (Table 2). Typical clinical PVCs were registered and captured.

The patient underwent cMRI (Magnetom Skyra 3 T, Siemens AG, Munich, Germany). A coronal T1_vibe_fat-sat sequence with a thickness of 2 mm, field of view (FOV) of 500 × 500 mm, and 160 images in the coronal plane before contrast medium administration allowed detection of the full anatomical torso with the applied carbon ECG strips (Figure 3D,E). The transversal T1_vibe_fat-sat sequence with a thickness of 1.2 mm, an FOV of 380 × 380 mm, and 144 images after intravenous administration of contrast medium (Gadovist 0.1 mmol/mL—6 mL) during a breath hold helped to acquire the full heart (Figure 3F).

Non-invasive, invasive, and fusion data analyses using the MRI data were performed in the same manner as in case N1. Invasive mapping of the LV was performed through foramen ovalis. The numbers of LV points used in the non-invasive and invasive mapping were 825 and 150, respectively.

The EAZ of a typical PVC was located on the inferoseptal apical part of the LV. Our invasive mapping demonstrated that the measured distance from the EAZ to the mitral annulus amounted to 73 mm. Similarly, the distance on the non-invasive map was 75 mm (Table 3).

The area of the EAZ was concordant between the invasive/non-invasive mapping (7.1 versus 7 cm^2^) (Table 3).

**Figure 7:**
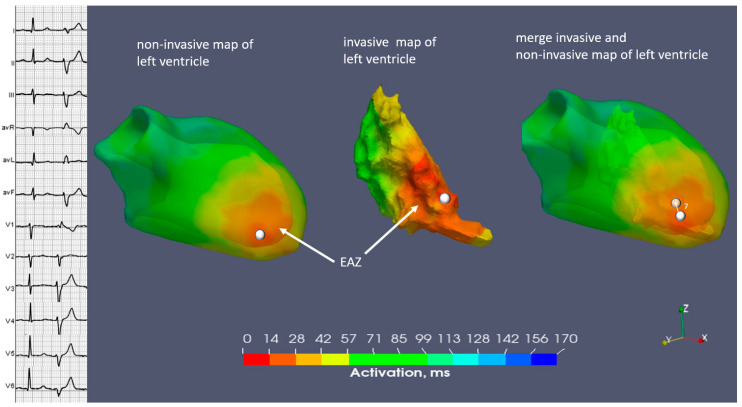
Case N2. Sinus rhythm, premature ventricular contraction. Three-dimensional representation of the endocardial non-invasive isochronal map on the left, of the invasive isochronal map in the middle, and the fusion of non-invasive and invasive maps on the right. EAZ—early activation zone. White marker—the earliest activation site of EAZ. Approximately 7 mm distance between non-invasive and invasive earliest activation site on fusion model.

The correspondence between these two methods was represented on a merged image with a difference of 7 mm (Figure 7). Successful endocardial ablation with no PVC recurrence at a six-month follow-up was achieved.

### 2.3. Case N3

A patient symptomatic for dyspnea on exertion during bigeminy and frequent PVCs without any known structural cardiomyopathy was referred to our cardiology department. Therapy with a beta-blocker and propafenone was not effective. The PVCs’ burden varied between 3 and 5%. A transthoracic echocardiography demonstrated preserved systolic function of the LV.

**Figure 8:**
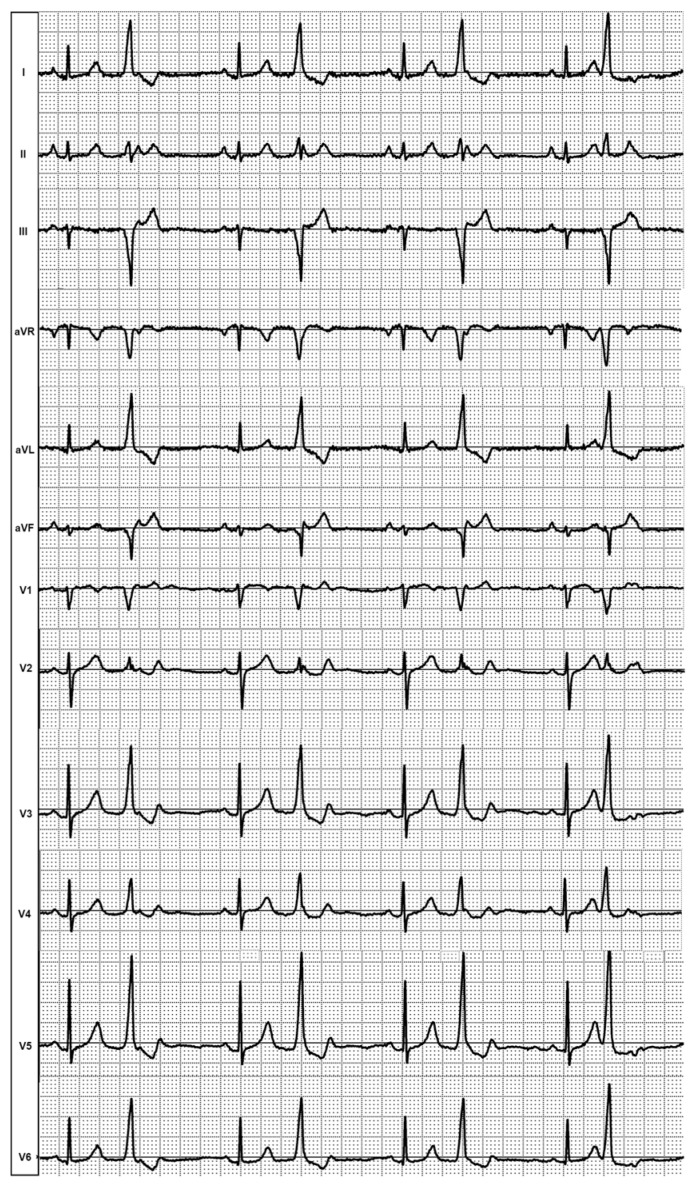
Case N3. Sinus rhythm, monomorphic premature ventricular contractions.

The 12-lead ECG demonstrated sinus rhythm and frequent monomorphic PVCs with a narrow QRS (120 ms), discordance in the QRS complex (positive in II and negative in III lead), Qr morphology in the V1 precordial lead, and right bundle branch block morphology in V2 (Figure 8).

The characteristics of the arrhythmia are presented in Table 1.

In case N3, 21 strips corresponding to 168 unipolar body surface CT-compatible electrodes were used for ECG recording during sinus rhythm (Table 2). Typical clinical PVCs were registered and captured.

The patient underwent cCT in the same manner as in case N1. A retrospective ECG-gated scan of the heart was performed with contrast medium administration (Ultravist 370—70 mL).

The non-invasive, invasive, and fusion data analyses were conducted in the same manner as in case N1. Both ventricular chambers were mapped during the invasive procedure. Invasive mapping of the LV was performed after transseptal puncture. The number of RV points used in the non-invasive and invasive mapping was 623 and 250, respectively.

On the non-invasive and invasive isochronal maps, the EAZ was located in the basal part of the right ventricle (inferoseptal, near the tricuspid annulus). The distance from the EAZ to the tricuspid annulus was 9 mm versus 12 mm on the invasive and non-invasive mapping, respectively. The area of the EAZ was 0.4 cm^2^ on the invasive and 0.6 cm^2^ on the non-invasive isochronal map, respectively (Table 3).

**Figure 9:**
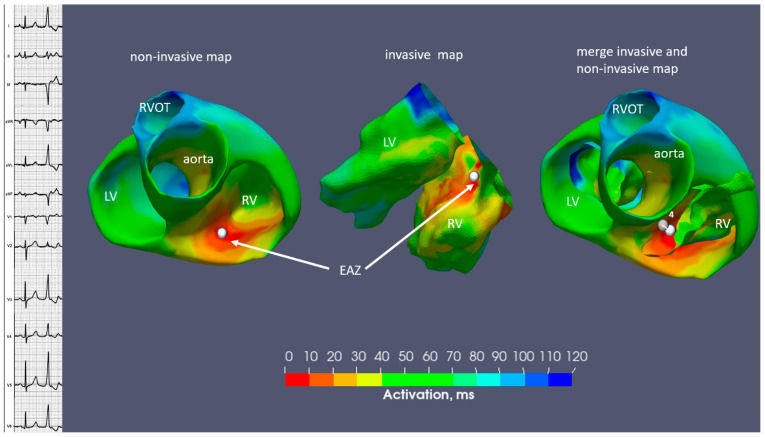
Case N3. Sinus rhythm, premature ventricular contraction. Three-dimensional representation of the endocardial non-invasive isochronal map on the left, of the invasive isochronal map in the middle, and the fusion of non-invasive and invasive maps on the right. EAZ—early activation zone; LV—left ventricle; RV—right ventricle; RVOT—right ventricular outflow tract. White marker—the earliest activation site of EAZ. Approximately 4 mm distance between non-invasive and invasive earliest activation site on fusion model.

The distance between the earliest activation site on the fusion model was 4 mm (Figure 9).

## 3. Discussion

A search of the literature exhibits a large number of works devoted to ECGI and invasive mapping, mainly of the endocardium and, to a lesser extent, of the epicardium in ventricular arrhythmias [7,9,10,14,15,16,17,18,20,21,22,23,24,25,26,27,28]. The majority of such works used the invasive 3D mapping CARTO system (Biosense Webster, Irvine, CA, USA) [7,8,9,10,14,15,16,17,18,19,20,21,22,23,24,25,26,27,28,29,30,31], which could be easily explained by its overwhelming market presence. However, there are also some other systems, such as EnSite NavX Precision (Abbott, Chicago, IL, USA), which we use daily in our work. To our best knowledge, this is the first time a series of cases has highlighted a qualitative and quantitative comparison of non-invasive ECGI (Amycard 01C, EP Solutions SA) with invasive mapping from EnSite NavX Precision, Abbott.

The used non-invasive mapping system is able to work with both contrast cCT and cMRI, so we could include both patients’ categories in the current analysis. Furthermore, our system’s choice was based on the fact that the system provides information not only about the epicardium, but also about the ventricular endocardium. This might be an important clinical component, since most ectopic beats have an endocardial origin.

While conducting the current investigation, we realized that it is a team effort that requires certain skills. A full-fledged clinical study requires the participation of at least one electrophysiologist and one radiologist involved in cardiac imaging.

The qualitative analyses in each of the three cases showed the same early localization of the dominant PVC on the endocardial surface when comparing the non-invasive and invasive isochronal maps. Our results are in line with earlier publications demonstrating the feasibility of the ECGI provided by the Amycard 01C system [14,15,16,17,18,25].

Novel in the current work is also the comparative quantitative analysis between technologies (invasive versus non-invasive). First, a similar measurement of the distance between the EAZ and atrioventricular annulus was obtained. In all three cases, the results were comparable between the invasive and non-invasive data (36/41 mm in case N1, 73/75 mm in case N2, 9/12 mm in case N3). Invasively, the location of the annulus was established based on the electrical signals typical for the annular zone and not anatomically, as during the ECGI. This may be the cause of the small differences observed between the two technologies. Second, the area of EAZ was also similar between the invasive/non-invasive maps (4.3/4.5 cm^2^ in case N1, 7.1/7.0 cm^2^ in case N2, 0.4/0.6 cm^2^ in case N3). To our knowledge, this is the first study showing such quantitative comparison.

Furthermore, a fusion of both the non-invasive and invasive isochronal maps was performed in order to evaluate the concordance between the two technologies in localizing the EAZ in patients with PVCs. Similarity between the earliest activation site on the non-invasive and invasive isochronal maps was quite high (4 mm in case N1, 7 mm in case N2, 4 mm in case N3). It should be taken into account that the tip of an invasive catheter is 4 mm long with a diameter of 7 French, which is why the aforementioned accuracy between the maps can be considered high. It is also worth noting that the reported errors already include a potential mismatch between the cardiac positions in sinus rhythm (the source for cCT anatomical reconstructions) and PVC beats (the source for invasive mapping and electrocardiographic reconstructions).

All the presented quantitative measurements demonstrate the reliability of a non-invasive approach in order to plan and guide invasive treatment in the clinical practice. Particularly, a non-invasive assessment can be useful in cases of intermittent ventricular ectopias, as it seems to be sufficient to register a single beat for analysis and, therefore, to overcome difficulties during invasive ablation procedures in which the PVCs are neither present nor inducible. Furthermore, this technology could be useful in complex PVC cases originating from the LVOT/RVOT areas, where ECG interpretation might be ambiguous.

## 4. Conclusions

This is the first case series with PVCs to demonstrate a qualitative and quantitative comparison of non-invasive ECGI (Amycard 01C, EP Solutions SA) with invasive mapping (EnSite NavX Precision, Abbott).

The distance from the EAZ to the annulus and the EAZ area as parameters for matching isochronal maps were similar when comparing the non-invasive and invasive models.

The similarity between the earliest activation site on the non-invasive and invasive isochronal maps was sufficient and appropriate to trust the preoperative analysis in these cases. Although these findings lack statistical power, the presented case results encourage further investigations.

## Figures and Tables

**Table 1:** Arrhythmias’ data.

	Case 1	Case 2	Case 3
Cardiac CT/MRI	cCT	cMRI	cCT
Structural cardiomyopathy	Ischaemic (previous inferior wall infarction)	None (aortic valve insufficiency)	None
EF LV (%)	29	48	64
PVCs burden % on a Holter monitors	20–50	30	5
PVCs morphology	Monomorphic	Monomorphic	Monomorphic
12 lead ECG	Superior axis; RBBB-morphology; R/S transition in V5-V6	Superior axis; RBBB-morphology; R/S transition in V1-V2; equiphasic complex in I lead	Discordance in QRS complex (positive in II, negative in III); positive in I; narrow QRS 120 ms
Antiarrhythmic therapy	Beta-blockers	Beta-blockers, Flecainid, Propafenone	Beta-blockers, Propafenone

**Table 2:** Number and material of ECG strips used for each case.

	Number of 8-Electrode ECG Stripes	Number of Unipolar Electrodes	Electrode Material
Case 1	26	208	Metal
Case 2	28	224	Carbon
Case 3	21	168	Metal

**Table 3:** Results of invasive and non-invasive mapping.

	Case 1	Case 2	Case 3
Position of EAZ			
Invasive	inferior wall LV	inferoseptal apical LV	inferoseptal basal RV
Non-invasive	inferior wall LV	inferoseptal apical LV	inferoseptal basal RV
Area of EAZ, cm^2^			
Invasive	4.3	7.1	0.4
Non-invasive	4.5	7	0.6
Distance from EAZ to annulus, mm			
Invasive	36	73	9
Non-invasive	41	75	12
Distance between the earliest activation site on invasive and non-invasive map, mm	4	7	4

## Data Availability

This data can be found here: [https://drive.google.com/file/d/1RxkEwPmu7V23ByMHJmObs5CGW2hxolzb/view?usp=sharing].

## Abbreviations

EAZ: early activation zone; ECG: electrocardiography; ECGI: non-invasive electrocardiographic imaging; EF: ejection fraction; LV: left ventricle; PVCs: premature ventricular contractions; RBBB: right bundle branch block; cCT: cardiac computer tomography; cMRI: cardiac magnetic resonance.
